# Management of gynecological cancers in the COVID-19 era: a survey from Turkey

**DOI:** 10.4274/jtgga.galenos.2020.2020.0071

**Published:** 2020-12-04

**Authors:** Duygu Altın, İbrahim Yalçın, Ghanim Khatib, Mine Dağgez Keleşoğlu, Sedat Akgöl, Ayşe Büşra Önder, İlker Kahramanoğlu, Tevfik Güvenal, Samet Topuz, Fuat Demirkıran

**Affiliations:** 1 Clinic of Obstetrics and Gynecology, Ordu University Training and Research Hospital, Ordu, Turkey; 2 Clinic of Obstetrics and Gynecology, Şanlıurfa Training and Research Hospital, Şanlıurfa, Turkey; 3 Department of Obstetrics and Gynecology, Çukurova University Faculty of Medicine, Adana, Turkey; 4 Department of Obstetrics and Gynecology, Erciyes University Faculty of Medicine, Kayseri, Turkey; 5 Clinic of Obstetrics and Gynecology, University of Health Sciences Turkey, Kanuni Sultan Süleyman Training and Research Hospital, İstanbul, Turkey; 6 Clinic of Obstetrics and Gynecology, Bakırköy Dr. Sadi Konuk Training and Research Hospital, İstanbul, Turkey; 7 Department of Obstetrics and Gynecology, İstanbul University-Cerrahpaşa, Cerrahpaşa Faculty of Medicine, İstanbul, Turkey; 8 Department of Obstetrics and Gynecology, Manisa Celal Bayar University Faculty of Medicine, Manisa, Turkey; 9 Department of Obstetrics and Gynecology, İstanbul University İstanbul Faculty of Medicine, İstanbul, Turkey

**Keywords:** COVID-19, gynecologic oncology, survey

## Abstract

**Objective:**

This study aimed to investigate how gynecologic oncologists modified their patient management during Coronavirus disease-2019 (COVID-19) in Turkey.

**Material and Methods:**

An online survey was sent to gynecologic oncology specialists and fellows in Turkey. It included management questions about strategies for newly diagnosed or recurrent endometrial, cervical, ovarian and vulvar cancer during the pandemic. Participants were asked if treatment of these cancers can be delayed or not and, if yes, the duration of delay.

**Results:**

32.9% of surgeons prescribed oral or intrauterine progesterone for early stage, low-grade endometrial cancer. Conversely, 65.7% and 45.7% of the most surgeons did not change their management for early stage high-grade and advanced stage endometrial cancers respectively, as they perform surgery. 58% and 67.1% of the surgeons continued to prefer standard surgical treatment for microinvasive and early stage cervical cancers, respectively. Radiotherapy was preferred administered with hypofractionated doses for locally advanced cervical cancer (57.1%). While 67.1% of surgeons operated early stage ovarian cancer patients, 50% administered neoadjuvant chemotherapy (NACT) to all advanced stage ovarian cancers and 50% administered more cycles of NACT in preference to interval debulking surgery. 93.7% of the surgeons responded that treatment should not be delayed beyond eight weeks.

**Conclusion:**

Most Turkish gynecologic oncologists modified their management of gynecologic cancers due to the COVID-19 pandemic. While chemotherapy was preferred for ovarian cancer, postponement of the surgery, with or without non-surgical options, was considered for early stage, low-grade endometrial cancer. Treatment of gynecologic cancers should be decided on a case by case basis, taking into account local COVID-19 infection rates and availability of health facilities. Prognosis is also an important consideration if delay is contemplated. Standard treatment and normal time-frames should be used if possible. If not, a postponement for a maximum of eight weeks or referral to another center were acceptable alternatives.

## Introduction

At the end of 2019, a novel type of coronavirus, Severe Acute Respiratory Syndrome-Coronavirus-2 (SARS-CoV-2), was identified as the cause of severe pneumonia in China (1). Since then, with the rapid spread of the Coronavirus disease-2019 (COVID-19) and almost all the countries of the world being affected, the World Health Organization defined the disease as a pandemic in March 2020.

In many countries, most hospital beds were occupied by COVID-19 patients, specialists from all branches were assigned to assist COVID-19 patients and elective surgeries have been limited. The management of cancer patients under these circumstances is controversial. It has been reported that cancer patients are more susceptible to COVID-19 (2). However, delay in treatment may worsen prognosis and chance of cure. Thus the main objective has become to treat cancer patients as quickly as possible while limiting the risk of infection.

Like all cancer patients, gynecologic cancer patients should continue to receive health care during the pandemic. However, clinical management has become more challenging for surgeons, since blood products or intensive care unit (ICU) beds may not be available due to COVID-19. Many organizations and associations issued new guidelines, taking into account the effect of the pandemic, for the management of gynecologic cancers (2,3).

As of 10 May 2020, 138,657 cases of COVID-19 have been reported in Turkey and cases continue to occur with variable incidence. The Turkish Society of Gynecologic Oncology (TRSGO) has issued its recommendations that management of gynecological cancer patients may differ between centers according to available resources (4).

The aim of this study was to investigate how gynecologic oncologists modified their management of gynecologic malignancies during the COVID-19 pandemic in Turkey.

## Material and Methods

This study was approved by Ordu University Institutional Review Board (approval number: 2020/77). A questionnaire developed by the TRSGO was sent to gynecologic oncology specialists and trainees working actively at either university hospitals, training hospitals, public hospitals or special clinics across Turkey via the internet. The survey was sent in April 2020, along with an informed consent form. Respondents were able to complete and return the survey online. The questionnaire included how management of endometrial, cervical, ovarian and vulvar cancer changed during the pandemic. Participants were also asked if treatment of these cancers can be delayed or not and, if yes, the duration of delay.

### Statistical analysis

Descriptive analyses are presented as numbers and percentages. Chi-square test and Fisher’s exact test were used to compare clinico-pathologic characteristics. Values of p<0.05 were considered statistically significant. SPSS, version 21.0 (IBM Inc., Armonk, NY, USA) was used for statistical calculations.

## Results

The survey was sent to 172 physicians listed in the TRSGO database. Of these, 70 (40.7%) gynecologic oncologists or fellows in gynecologic oncology answered the survey. As seen in Table 1, most of the participants were consultants (n=55, 82.1%) and working at either university or training hospitals (n=58, 82.8%). Almost all of them stated their management had changed after the pandemic and they preferred laparotomy (L/T) to laparoscopy (L/S) (73.9% vs 26.1%). While 27 (38.6%) participants believed the risk of getting infected by COVID-19 was more than 20%, 14 (20%) thought it was less than 5%. The majority of the surgeons (n=49, 70%) expect to get back to normal in 2-5 months.

### Endometrial cancer

Table 2 shows the approach to patients who are newly diagnosed with endometrial cancer after COVID-19. While most surgeons delayed the surgery (20%) and preferred medical treatment, either with intrauterine or oral progesterone (32.9%) for early stage low-grade endometrial cancer, staging surgery (65.7%) continued to be the mainstay treatment of early stage, high-grade (grade 3/serous/clear cell, etc.) endometrial cancers. Most surgeons continued to perform standard debulking surgery (45.7%) for advanced stage endometrial cancer but 32.9% chose to administer chemotherapy (CT) instead of surgery during the pandemic.

### Cervical cancer

Table 2 shows the approach to cervical cancer patients who are newly diagnosed or in whom disease has recurred after COVID-19. While most surgeons continue to operate (58%) microinvasive cervical cancer, 33.3% delayed the surgery. Likewise, standard surgery (67.1%) and delay (20%) were the two leading responses when asked about their approach to early stage cervical cancer. Primary radiotherapy (RT) or chemo-RT was applied without delay to most of the locally advanced cervical cancer (LACC) patients, but hypo-fractionation of the dose (57.1%) was preferred to standard dose (27.1%), in order to reduce the number of hospital visits. 67.1% of surgeons continued to perform exenterative surgery or administered CT/RT to metastatic or recurrent cervical cancer patients.

### Ovarian cancer

67.1% of participants did not change their management (staging surgery) in early stage ovarian cancer. If it was not possible to operate, they mostly (12.9%) referred patients to more suitable cancer centers, rather than administering CT after obtaining tissue biopsy. While 38.6% continue to perform interval debulking surgery (IDS) for patients who had already completed their neoadjuvant chemotherapy (NACT), 50% administered more cycles of CT and 11.4% referred patients to another center. 50% of surgeons administered NACT to all advanced stage ovarian cancers, 20% continued to operate, 17.1% limited cytoreductive surgery indication and the remainder either delayed the operation or referred the patients elsewhere. 32.9% of surgeons administered NACT according to cytology, 15.7% performed diagnostic L/S, and 48.6% referred patients to interventional radiology for tissue biopsy. 2.9% administered NACT if there was a very high suspicion of ovarian cancer without confirmation by cytology or tissue biopsy. While 44.3% continued to operate recurrent ovarian cancer patients who were suitable for surgery, 28.6% administered CT and the rest either delayed the operation or referred the patients elsewhere.

### Vulvar cancer

As seen in Table 3, most surgeons either preferred to perform surgery immediately (61.4%) or delay it for a couple of weeks (27.1%) for newly diagnosed, early stage vulvar cancer patients. For advanced stage vulvar cancer, most surgeons (64.3%) did not change their practice.

Participants were asked to score their priority for treatment of each gynecologic cancer from 1 to 5, with 1 the lowest priority and 5 the highest priority. One participant did not answer this part of the questionnaire. Table 4 shows the results.

While continuing to be mindful of disease progression, participants were asked their opinion on the maximum time (in weeks) that treatment can be delayed under COVID-19 conditions. As seen in Table 5, most surgeons thought that treatment should start within eight weeks of diagnosis. It was also evident that respondents believed that treatment should start earlier in advanced stage and/or high-grade cancers compared to early stage and/or low-grade cancers. There were significant differences between answers concerning low- and high-grade early stage endometrial cancers (p<0.001), early and advanced stage endometrial cancers (p=0.024), early stage and LACC (p<0.001), early and advanced stage ovarian cancers (p=0.039), and early and advanced stage vulvar cancers (p=0.014).

## Discussion

With the rapid spread of COVID-19 throughout the world, national health systems of many counties experienced additional stresses. Many countries, including Turkey, took steps to slow the spread of infection. Much elective surgery was suspended to allow resources to be deployed for COVID-19. Despite this situation, it was apparent that clinicians had a duty to continue to provide health care to gynecologic oncology patients.

97.1% of responding Turkish gynecologic oncologists stated their cancer management changed during the pandemic. Surgical treatment remained the gold standard for many types of gynecologic cancers. However, performing surgery may not be possible under pandemic conditions. There are several reasons for this. First, gynecologic oncology patients are generally old and have pre-existing comorbidities such as cardiovascular disease or diabetes mellitus. Therefore, they may need observation in ICU postoperatively. Even a young patient undergoing radical surgery and multiorgan resection may need ICU. Unfortunately, many ICU beds were, and continue to be, occupied by COVID-19 patients. Second, some hospitals ran out of blood and blood products since fewer people made blood donation due to the pandemic. Third, when COVID-19 is more prevalent, COVID-19 patients are hospitalized not only in infectious disease or respiratory disease clinics, but also in any available hospital bed. Many clinics were converted into COVID-19 clinics due to the growing number of infected patients. Lastly, some gynecologic oncologists were recruited to take care for COVID-19 patients (5,6).

The route of surgery was another change during the pandemic. The majority of surgeons (73.9%) preferred L/T rather than L/S because of concern about viral transmission via contaminated aerosol produced from port sites during L/S. To date, there is no evidence to show that the COVID-19 virus speads via laparoscopic smoke plume and this theoretical risk was extrapolated from other viral infections. For example, human immunodeficiency virus (HIV), hepatitis B virus (HBV) and human papillomavirus (HPV) have been detected in surgical smoke (5,6,7,8). Although there are a few cases reporting HPV transmission via surgical smoke, HIV and HBV transmission have not been documented (7,9). In light of this theoretical risk surgeons preferred L/T to minimize exposure to these blood borne pathogens, when possible, although no studies have identified COVID-19 in surgical smoke nor reported transmission of other coronaviruses through surgical smoke.

Performing operations solely using L/T because of this hypothetic risk may result in more surgical complications, such as blood loss, wound infection or atelectasis, longer hospital stay and greater risk of COVID-19 exposure for the patient. Since electrosurgical devices create surgical smoke plumes, they potentially increase viral transmission both in open and minimally invasive surgeries. There is no evidence to prove that infection occurs more often via L/S compared to L/T. Nevertheless, precautions should be taken to minimize this theoretical risk and these should include all operating room personnel being equipped with adequate personal protective equipment, L/S being performed with lower intra-abdominal pressure when possible, use of energy should be minimized and smoke evacuation/filtration should be used (8,10). Therefore, we believe that surgeons should decide the route of surgery on a case by case basis.

Oral progesterone or progesterone releasing IUD is the most common management strategy for early stage, low-grade endometrial cancer amongst Turkish gynecologic oncologists and it is a reasonable alternative to surgery. Several studies have reported 75-80% regression rate with oral progestins in young endometrial cancer patients who want to retain fertility (9,10,11,12). Even without progesterone therapy, surgery can be postponed for 1-2 months for low-risk endometrial cancers without loss of cure chance (2). Therefore, surgeons prefer to delay surgery, even if they have available resources for the operation. Conversely, most surgeons continue to operate early stage, high-grade and advanced stage endometrial cancer, since the patient may not be cured as a result of treatment delay. Although only 7.1% of respondents preferred it, simple hysterectomy and bilateral salpingo-oophorectomy ± sentinel lymph node biopsy is another option for high-grade endometrial cancer treatment, as reported by Ramirez et al. (3) that has been shown to reduce operative morbidities.

Surgery is the accepted standard of care for early stage cervical cancer. As such, most Turkish gynecologic oncologists continue to operate microinvasive and early stage cervical cancers. If immediate operation was not possible, operations were delayed, since postponing the surgery for 6-8 weeks has been recommended as acceptable for localized disease during the pandemic (3). Primary (chemo) RT was being instigated without delay for most LACC patients, but hypofractionation of the dose (57.1%) was preferred to standard dose (27.1%), in order to reduce the number of hospital visits.

67.1% of participants perform staging surgery for early stage ovarian cancer. Performing only adnexectomy and deferring the staging surgery for 1-2 months may be another alternative under pandemic conditions (2). The benefit of upfront surgery in advanced stage ovarian cancer is well known and NACT is administered for certain indications (11,13). Despite this, half of Turkish gynecologic oncologists, in keeping with their colleagues worldwide, administer NACT to all patients (12,6). Only 20% perform cytoreductive surgery. The availability of ICU beds and blood products may be the reasoning behind this choice, since the majority of these patients need multiorgan resections. 50% of surgeons administered more than 3-4 cycles of NACT before the IDS, as recommended (2,3). One of the remarkable results of this survey was that 48.6% of surgeons referred patients to an interventional radiologist for tissue biopsy before initiation of NACT. Only 15.7% performed diagnostic L/S, which may be a consequence of concerns about viral transmission during L/S. Although ascites cytology is highly accurate in diagnosing ovarian cancer, 32.9% administer NACT according based on the results of cytology (13,14,15).

The majority of surgeons treated both early and advanced stage vulvar cancer as they had done prior to the pandemic. This result is unsurprising since surgery is the only treatment option in many cases. When the tumor is small, it has been reported that it is acceptable to postpone surgery for a couple of months (2).

Time is particularly important in the fight against cancer as the chance of achieving a cure can be lost if if the delay is too long. However, due to the pandemic, delay in treatment is currently unavoidable. Therefore, one should always keep in mind which patients will benefit most from treatment, who needs to be treated urgently and who can wait for some time without disease progression. Participants indicated a general belief that timing of treatment is more important for advanced stage and high-grade tumors compared to early stage and low-grade tumors. Hence, a delay in cancer treatment to minimize infection risk is more commonly associated with early stage cancers compared to advanced stage tumors.

This is the first national survey of gynecologic oncologists regarding changes to their practice due to the COVID-19 pandemic. A strength of this study is the inclusion of an homogenous group of gynecologic oncologists who actively work in Turkey. However, as this is a single country survey and only 40.7% of gynecologic oncologists responded, it may limit generalization.

## Conclusion

Most gynecologic oncologists have changed their management for gynecologic cancers due to the pandemic. While surgery was postponed and progesterone treatment was preferred in early stage low-grade endometrial cancer, CT came to fore for ovarian cancer. Surgery is performed immediately or with a delay for microinvasive and early stage cervical cancers and hypofractionated dose is preferred for LACC. The number of COVID-19 infected patients and availability of health facilities differ from center to center within Turkey. During the pandemic, treatment for gynecologic cancers should be decided on a case by case basis, taking into account local resource availability and local risk of infection, in conjunction with the chance of achieving a cure. The authors believe that, applying standard treatment when possible and, if not, postponing the treatment for a couple of months in patients in whom it is safe to do so or referral to another cancer center when delay is inadvisable are the best choices at the current time.

## Figures and Tables

**Table 1:**
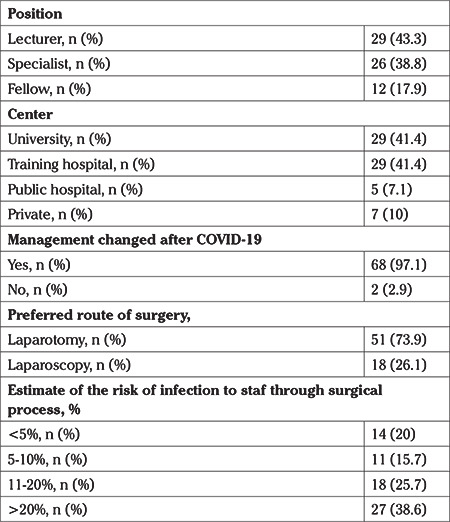
Characteristics of participants

**Table 2:**
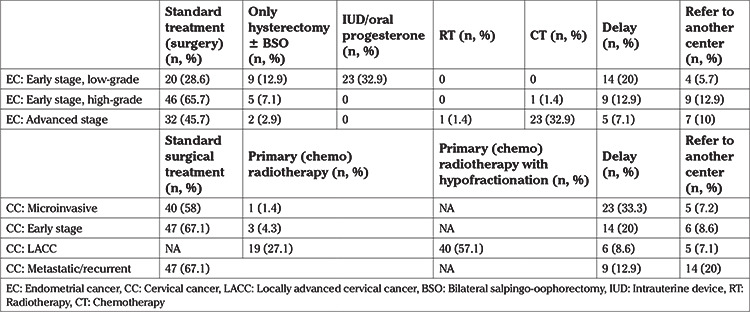
Management of newly diagnosed endometrial cancer and newly diagnosed or recurrent cervical cancer patients after the pandemic

**Table 3:**
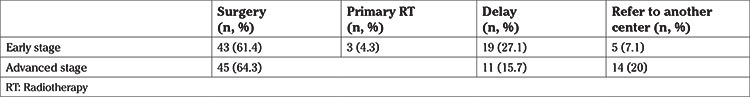
Management of newly diagnosed vulvar cancer patients after the pandemic

**Table 4:**
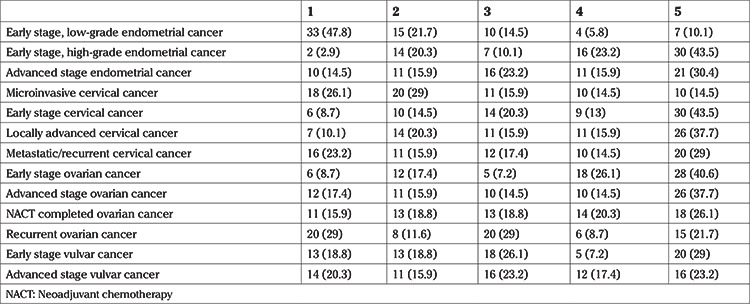
Priority of treatment during the pandemic. 1 = lowest priority and 5 = highest priority

**Table 5:**
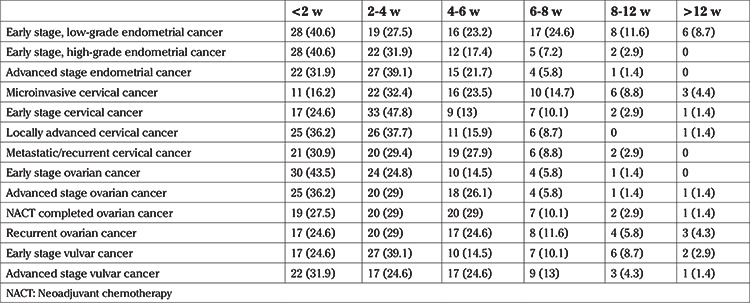
Responses to the question “How long can treatment be delayed during the pandemic?” Data are given as (n, %)
